# MIXED-METHODS RESEARCH DESIGNS FOR EARLY-PHASE DYADIC HEALTH RESEARCH

**DOI:** 10.1093/geroni/igae098.1866

**Published:** 2024-12-31

**Authors:** Michael McCarthy, Eric Cerino, Megan McCoy, Margarita Martinez

**Affiliations:** Northern Arizona University, Flagstaff, Arizona, United States; Northern Arizona University, Flagstaff, Arizona, United States; Northern Arizona University, Northern Arizona University, Arizona, United States; Northern Arizona University, Northern Arizona University, Arizona, United States

## Abstract

Individuals and family caregivers living in rural areas face a double bind when it comes to health. First, they may be at increased risk for chronic health conditions. Second, they are frequently hampered in seeking support for these conditions due to factors including geographic isolation, cultural barriers, and lack of financial resources. These inequities result in delayed diagnosis and treatment and poor health outcomes. Researchers seeking to address these issues are often faced with recruitment challenges in small rural communities and subsequently modest sample sizes. Mixed-methods designs offer advantages for early-phase dyadic studies including strengthening participant engagement, maximizing resources, surfacing new questions to be explored in future research, and in general, providing a deeper understanding of the phenomenon being studied. In this presentation, we draw on several of our mixed-methods early-phase dyadic studies in stroke, dementia, and cognitive health to provide examples. We identify mixed-methods designs that deepen understanding in early-phase dyadic health research and the advantages of certain mixed-methods designs for certain inquiries. Finally, we discuss recommendations for maximizing the approach, and strategies for blending analytical methods.

